# Effects of social factors on the COVID-19 cases and its evolution in Hubei, China

**DOI:** 10.3389/fpubh.2023.1124541

**Published:** 2023-06-16

**Authors:** Shuqi Yin, Lijing Du, Dongmei Meng

**Affiliations:** ^1^School of Management, Research Institute of Digital Governance and Management Decision Innovation, Wuhan University of Technology, Wuhan, Hubei, China; ^2^School of Management, Lanzhou University, Lanzhou, Gansu, China; ^3^Tianjin Children Hospital, Tianjin, China

**Keywords:** evolution of the COVID-19, social factors, time-series data, lag period, linear model, statistical analysis

## Abstract

**Introduction:**

In order to study the impact of social factors on the evolution of the epidemic, this paper takes the COVID-19 in Hubei Province of China as an example to study the impact of social factors such as the permanent population, universities, hospitals, the distance between Wuhan seafood market and 17 cities in Hubei Province, and the distribution of medical supplies on the COVID-19. This is of great significance for helping to develop effective prevention and control measures and response strategies, ensuring public health and social stability.

**Methods:**

Time series regression analysis is used to study the impact of various factors on the epidemic situation, multidimensional scale analysis is used to assess the differences among provinces, and Almon polynomial is used to study the lag effect of the impact.

**Results:**

We found that these cities can be divided into three groups based on the number of confirmed cases and the time course data of the cases. The results verify that these factors have a great impact on the evolution of the COVID-19.

**Discussion:**

With the increase in the number of universities, the number of confirmed cases and new cases has significantly increased. With the increase in population density, the number of new cases has significantly increased. In addition, the farther away from the Wuhan seafood market, the fewer confirmed cases. It is worth noting that the insufficient increase in medical supplies in some cities still leads to a significant increase in new cases. This impact is regional, and their lag periods are also different. Through the comparison with Guangdong Province, it is concluded that social factors will affect COVID-19. Overall, promoting the construction of medical schools and ensuring the reasonable distribution of medical supplies is crucial as it can effectively assist decision-making.

## 1. Introduction

As of May 20, 2022, according to reports, 523,431,796 cases of COVID-19 have been confirmed worldwide, 495,381,216 cases have been cured and discharged from hospital, and 6,281,384 people have died. COVID-19 had affected our lives significantly. COVID-19 had a great impact on the British beef and sheep supply chain—the overnight closure of hospitality and catering and the redirection of supplies to the retail sector, and the public health and economic system faced serious negative effects (1, 2). The prevention and control of COVID-19 was still grim.

Social factors played a decisive role in the prevention and control of infectious diseases (3). As early as 2005, WHO defined the social determinants of health as follows: in addition to those factors that directly lead to disease, the living and working environment and other factors that affect health are determined by people’s social status and resources. At the beginning of the outbreak of COVID-19, a serious shortage of medical resources occurred in Wuhan, Hubei Province. On January 31, 2020, Hubei Daily reported that there were only two specialized infectious disease hospitals in Wuhan with just less than 1,000 beds available, and medical materials were stretched. However, the influencing mechanisms of social factors on the evolution of COVID-19 were still obscure. In this paper, we chose Hubei as the case to investigate the impact of social factors on COVID-19. Here the social factors refer to the economic factors, the living environment, and medical factors. Economic factors imply economic development, measured by GDP, the poverty rate, and other factors. The living environment factors include temperature and social distance. The medical factors represent the medical condition of the research object, including the number of hospitals and the number of medical materials.

The research in this paper aims to answer the following three questions (RQs):

RQ1: Which of the above factors uniquely affected the evolution of COVID-19? How did they affect the cases of COVID-19?

RQ2: What was the impact of time-series medical supplies on COVID-19?

RQ3: Through the analysis of the investigated factors, what suggestions could be brought to the policy-making for the prevention and control of COVID-19?

So far, some scholars have conducted in-depth research on the impact factors of COVID-19. We have combed the recent relevant literature and divided these factors into three categories: medical resources, social variables, and environmental factors, as shown in Table 1.

**Table 1:** Research on influencing factors of COVID-19.

	Method	Related factors	Subjects	Author (year)
Economic factors	LR	GDP; race	Cities with a population of more than 1 million or metropolitan areas	Cao et al. (4)
	LRM	Unemployment; poverty population density	5,698 patients at Michigan Medical University	Gu et al. (5)
	RMP	SVI	U.S.A	Freese et al. (6)
Living environment factors	ANN	Social distancing (lockdown); Density of the population; Urban population; Gender ratio	Globality	Sharma Asha (7)
	QR	Obesity; population over the age of 65	184 countries	Ashish Upadhyaya (8)
	LR	Geographical location; temperature; humidity; wind speed	Cities or metropolitan areas with a population of more than 1 million	Cao et al. (4)
	Poisson, ZIP, ZINB	Temperature; humidity; wind speed	Kingdom of Saudi Arabia	Anam (9)
	Partial Correlation, Linear Mixing	Temperature factors at different income levels	Globality	Mizanur et al. (10)
	LR	Number of McDonald’s and fitness centers	New Jersey	Amaratunga (11)
	GWR	Air pollutants; meteorological factors	China	Pei Lin et al. (12)
	Regression	Sunrise distance; effective reproduction number	Switzerland	Lison et al. (13)
	LR	Latitude; longitude; temperature	Cities with a population of more than 1 million or metropolitan areas	Cao et al. (4)
	ITSA	Social distance	28 European countries	Vokó and Pitter (14)
	EM	Existing medical conditions; lack of water	Mexico	Revollo-Fernández et al. (19)
Medical factors	LRB	Coronary heart disease (CHD); cancer; antiviral drugs	Kurdistan Province	Eghbal (15)
	RAC	Age; sex; symptoms and hospitalization	In Regensburg, Germany	Lampl et al. (16)
	OLS; RCS	Number of inpatients in an intensive care unit	Italy	Lorenzoni et al. (18)

SVI, Social Vulnerability Index; QR, Quantile Regression; RCS, Restricted Cubic Spline; ITSA, Interrupted Time Series Analysis; LR, Logistic Regression; MLR, Multivariable Logistic Regression; MPR, Multivariable Poisson Regression; BLR, Binary Logistic Regression; OLS, Ordinary Least Square; ANN, Artificial Neural Network; ZIP, Zero-Inflated Poisson; ZINB, Zero-Inflated Negative-Binomial; GWR, Geographically Weighted Regression; EM, Econometric Model; RAC, Retrospective Analysis of a Cohort.

Economic and living conditions had been demonstrated to affect the evolution and development of COVID-19 (4). The urban ratio, population density Sharma (7), temperature, humidity, and wind speed affected the evolution and the number of COVID-19 (4, 9). In addition, in economies with different income levels, the impact of temperature on COVID-19 mortality was different. A warm climate may reduce mortality in high-income economies, but in low-income and middle-income countries, high daily temperature changes may increase mortality (10). The increase in the number of McDonald’s led to a decrease in the number of cases and deaths in New Jersey, while the number of fitness centers was related to the increase in the number of cases and deaths (11). Vokó and Pitter used interruption time series analysis to study the impact of social distance on COVID-19, identified the most likely change-points in 28 European countries, and confirmed that the “stay-at-home” national policy had made a meaningful contribution to the suppression of the European COVID-19 (14). There was a direct relationship between the rapid growth of inpatients in Intensive Care Units (ICUs) in Italy and mortality. The increase in daily ICU admissions resulted in a significant increase in mortality after 3, 7, and 14 days Lorenzoni et al. (18).

The above literature mainly analyzed the impact of population density, GDP, temperature, wind speed, social distance, number of inpatients, and other factors on COVID-19 through the Artificial Neural Network, Time Series Analysis, Logistic Regression, Geographical Weight, and other regression models. The review of the existing technology showed that economic, environmental, and medical factors have a direct impact on COVID-19. Given the importance of the influencing mechanisms of special social factors on the evolution of COVID-19, we studied how the social factors affected the evolution of COVID-19 in the cities of Hubei Province in China. We selected economic factors (GDP), environmental factors (the number of permanent residents, the number of universities, the distance to the South China Seafood Market), and medical factors (the number of hospitals, and the distribution of medical materials) as research variables. This study discussed the relationship between COVID-19 and the above factors through the regression analysis and analyzed the relationship between the distribution of medical materials in Hubei Province in March 2020 and the number of COVID-19 cases through time series.

This work contributed in three ways. Firstly, through discussion and analysis, the importance of the influencing factors in the evolution of COVID-19 in Hubei Province was proposed. Secondly, the impact of medical materials on COVID-19 and their lag periods were deeply analyzed, and a thought-provoking conclusion was found that the rational allocation of medical materials was of great importance for policy-making. More importantly, this paper judged the rationality of allocation of medical material through the above results, put forward targeted suggestions, minimized the waste of medical materials, and provided policy support for the government in the distribution of medical material and epidemic prevention and control.

The rest of the paper was structured as follows. In section “Methods”, we discussed the data sources and statistical methods used. In section “Results”, we gave the test results, evaluated the model, then discussed it in section “Discussion”, and draw a conclusion in section “Conclusions”.

## 2. Methods

### 2.1. Data source

#### 2.1.1. Case data

We collected the data of confirmed, dead, cured, suspected and new increased cases(confirm_add) in 17 cities such as Wuhan and Yichang, Hubei Province from January 21 to June 1 (15).^1^ Part of the data in Wuhan were shown in Table 2.

**Table 2:** Part of COVID-19 data in Wuhan in 2020.

Date	Confirm	Dead	Heal	Suspect	confirm_add
02.21	45,346	1,684	6,214	0	319
02.22	45,660	1,774	7,206	0	314
02.23	46,201	1,856	8,171	0	541
02.24	46,607	1,987	8,946	0	348
02.25	47,071	2,043	10,337	0	464
02.26	47,441	2,085	11,793	0	370
02.27	47,824	2,104	13,328	0	383
02.28	48,137	2,132	15,826	0	313
02.29	48,557	2,169	17,552	0	420
03.01	49,122	2,195	19,227	0	565

#### 2.1.2. Static social data

The data of the permanent resident population were from the statistical yearbook of Hubei Province in 2020^2^; the distance to the seafood market in Wuhan came from the distance calculation of the Gaude map; the number of colleges and universities came from the list of all colleges and universities in Hubei. Data were shown in Table 3.

**Table 3:** Data on social factors of each city.

	University/Institute	Permanent resident population/10,000	Hospital/Institute	Distance/km
Wuhan	86	1121.2	70	4.6
Xiaogan	4	492.1	31	44.1
Jingmen	2	289.75	12	201
Jingzhou	7	557.01	39	194
Huanggang	4	633.3	21	396.2
Ezhou	1	105.97	18	65.9
Yichang	3	413.79	24	284.7
Enshi	3	339	93	449.3
Suizhou	1	222.1	54	146.2
Xiantao	1	114.01	26	145.1
Huangshi	4	247.17	15	85.2
Xiangyang	4	568	98	259.2
Qianjiang	1	96.61	21	132.8
Shiyan	6	339.8	84	390.4
Shennongjia	0	7.61	1	388.9
Xianning	2	254.84	46	83.3
Tianmen	1	124.74	25	106.6

#### 2.1.3. Time series data of medical materials

The data on medical materials were from the announcement of distribution of protective materials in medical institutions in Hubei Province.^3^ Data were shown in Table 4.

**Table 4:** Data of medical materials in Wuhan in 2020.

Date	Medical protective clothing (MPC)	N95 face-mask (N95)	Medical surgical mask (MSM)	Medical protective face shield (MPFS)	Medical isolation gown (MIG)
02.21	14,971	15,330	31,349	1906	2,862
02.22	13,209	20,574	40,473	2,207	10,756
02.23	15,604	19,860	576,910	4,416	9,825
02.24	16,926	23,077	830,940	4,356	9,455
02.25	16,508	31,664	78,350	4,383	8,382
02.26	16,093	29,082	909,290	4,436	7,277
02.27	13,574	27,606	603,260	4,467	8,006
02.28	11,636	25,970	496,190	2,540	3,652
02.29	13,385	19,776	474,580	4,530	5,453
03.01	14,971	15,330	31,349	1906	2,862

### 2.2. Statistical analysis

In this study, we analyzed the total number of confirmed cases and time course data. The form of time course data were ($T_{i}$,$Y_{\mathrm{ij}}$), where $t_{i}$ was the date (*i* = 1, …, *m*), and $T_{\mathrm{ij}}$ was the total number of cases in City *j* up to the time $t_{i}$.

#### 2.2.1. Analysis of the number of cases

Since the number of cases was related to the population, this paper also used the proportion of cases in the county as the response variable, $Y_{j}=T_{\mathrm{ij}}/P_{j}$. In order to investigate the relationship between added cases and factors, it also used the added case $A_{\mathrm{ij}}$, $A_{ij}=T_{\left(i+1\right)j}-T_{ij}$, where $\;P_{j}$ was the population of City *j*. Due to the correlation between variables, this paper ran a linear regression model and then selected variables by reverse stepwise regression fitting. By the way, we first standardized the data to the range of 0 to 1 to avoid the influence of dimension.

#### 2.2.2. Explanatory variables

The explanatory variables of this study included the number of colleges $x_{1}$, the resident population $x_{2}$, the number of hospitals $x_{3}$, and the distance from the seafood market in Wuhan $x_{4}$.

In particular, the number of hospitals $x_{3}$ was closely related to the population $x_{2}$. Collinearity between independent variables would lead to invalid model construction and loss of effectiveness of regression fitting. To avoid this effect, the number of hospitals was divided by the total population to make the variables independent of each other. The matrices of scatter plots of the two datasets were shown in “Appendix A,” in which the variables were relatively independent.

#### 2.2.3. Data visualization

**Figure 1:**
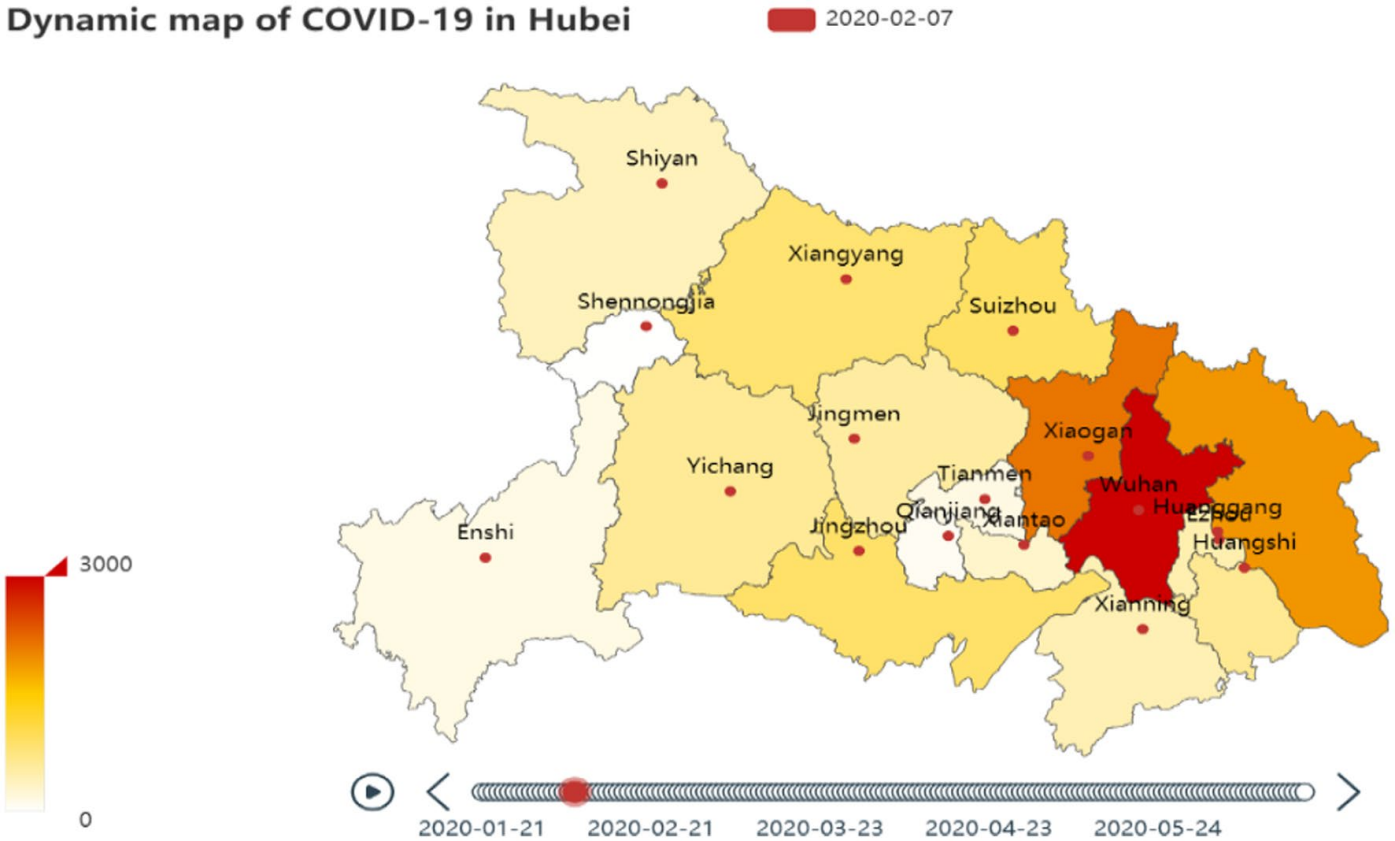
Dynamic map of COVID-19 in Hubei Province.

The visualization of COVID-19 data in Hubei Province could be drawn, as shown in Figure 1.

Embedding the data into the map of Hubei Province could realize the functions of viewing the specific quantity and changing the visual level through the mouse click, scroll wheel, suspend, and other operations, to avoid the problems of data loss and overlap. Figure 1 used color to distinguish the severity of COVID-19. According to the geographical distribution of the original diagnosis data, we could view the dynamic change trend and more intuitively analyze the evolution law of COVID-19 in Hubei Province.

#### 2.2.4. Dissimilarity matrix

The cumulative confirmed cases in cities in Hubei province continued to grow. In the early stage of COVID-19, the cases of cities increased exponentially, especially in Wuhan. Since then, the cumulative confirmed case curve of cities showed a horizontal state, indicating that the prevention and control of COVID-19 in Hubei Province had achieved remarkable results.

**Figure 2:**
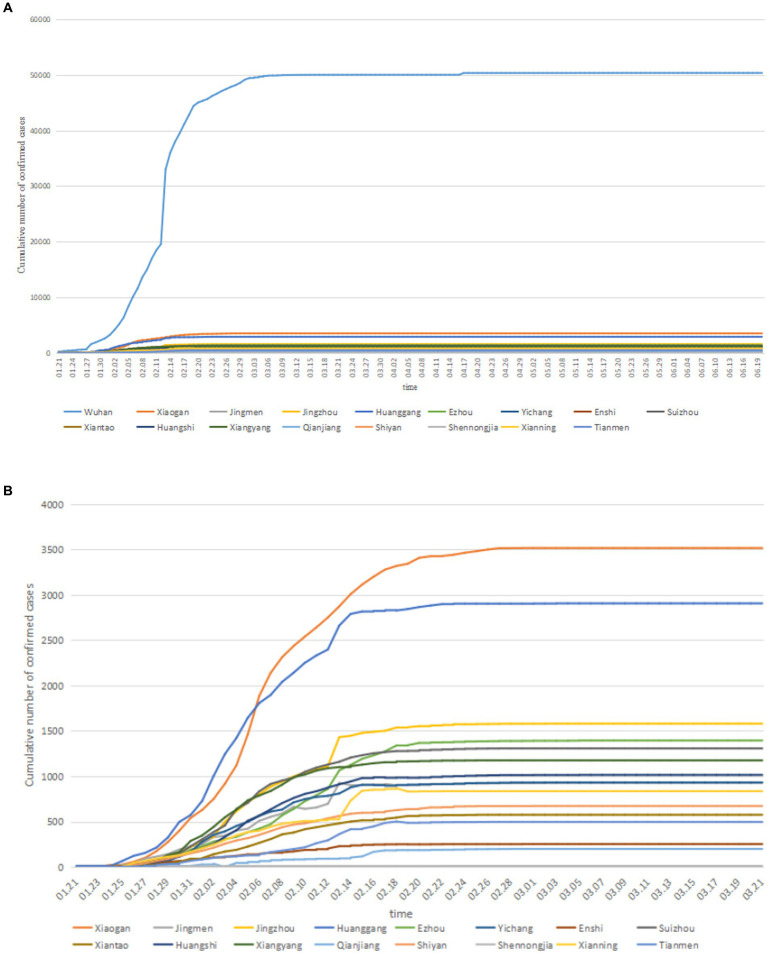
COVID-19 in Hubei **(A)** the trend map; **(B)** partial drawing (excluding Wuhan).

The time process data were shown in Figure 2. We used the area between city curves to define the difference $D_{AB}$ between City *A* and City *B*, using the following formula $D_{AB}=\sum_{i}\left|y_{iA}-y_{iB}\right|\delta_{i}$.

In this article, the horizontal axis represented the number of days, so $\delta_{i}=1$.

#### 2.2.5. Multidimensional scaling

**Figure 3:**
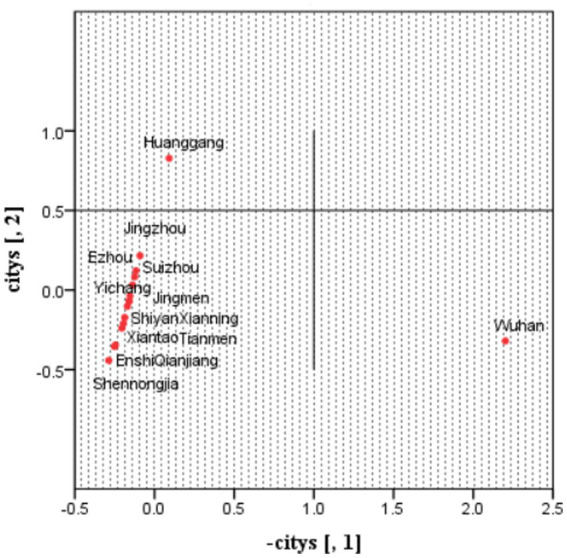
Scatter plots of MDS for the dissimilarity matrix relevant to the original case data.

Multidimensional scaling (MDS) can be used to find a set of points {$p_{A}$}, so that the Euclidean distance between $p_{A}$ and $p_{B}$ is close to $D_{AB}$. Since the first eigenvector of MDS can explain a large part of the differences between cities, it is reasonable to believe that the value {$p_{A}$} along the eigenvector carries the major information about the differences between counties. These values are expressed as {$p_{A}^{*}$}, then used as response variables and modeled according to explanatory variables. In this study, multidimensional scaling (MDS) generated the point set in Figure 3.

### 2.3. Time series data analysis

The explanatory variables included in this study include MPC $x_{5}$, N95 $x_{6}$, MSM $x_{7}$, MPFS $x_{8}$ and MIG $x_{9}$.

Firstly, the ADF test was selected to determine the stationarity of the sequence, and a differential method was used to realize the sequence stationarity of the non-stationary sequence. To alleviate the influence of heteroscedasticity, this paper reduces the scale of variables by taking the logarithm of time series. In order to avoid the quantity being 0, the model was constructed as follows:

$$\begin{array}{c} \ln\left(1+y\right)=C+\beta_{1}\ln\left(1+x_{5}\right)+\beta_{2}\ln\left(1+x_{6}\right)+\beta_{3}\ln\left(1+x_{7}\right)\\ \;\;+\beta_{4}\ln\left(1+x_{8}\right)+\beta_{5}\ln\left(1+x_{9}\right)+\delta \end{array}$$

The article then used the Almon method to calculate the lag period in the impact of medical supplies on new cases.

## 3. Results

### 3.1. Analysis of social factors

The regression results were shown in Table 5, where column T represented the total number of cases, column A represented the number of new cases, column T/P and column A/P represented the proportion excluding the impact of population. The effect was almost the same. The most important factor was the number of universities. As expected, with the increase of universities, the total number of cases and new cases have raised significantly. With the accretion of population density, the number of new cases increased significantly. Surprisingly, the data collected showed that the number of hospitals did not significantly affect the number of cases. On the other hand, the difference results of the proportion of cases (column T/P and column A/P) were similar to the results of the total number of cases (column T and column A), but the population had a great difference between column A and column A/P, indicating that the proportion of newly added cases increases significantly with the accretion of population density. GDP has no significant impact on the evolution of COVID-19 in cities of Hubei Province. It was worth noting that the distance from the seafood market in South China had a significant impact on the total number of cases, but has no significant impact on added cases.

**Table 5:** Regression results using the data.

Variables	T	T/P	A	A/P
University	0.06^&^	0.05^&^	0.17^&^	0.02^&^
Population	−0.01	−0.04	−0.12^^^	0.02^#^
Hospital/p	−0.29	−0.19	−0.76	−0.16
Distance	−0.04^#^	−0.01^&^		
GDP		0.04	−0.07	0.02

Significant codes: 0 &, 0.001 ^, 0.01 $, 0.05 #, 0.10.

### 3.2. Time series analysis of medical factors

The regression results of time series were shown in Table 6. The time series of cumulative medical factors had different effects on new cases in each province. Among them, Xiaogan and Enshi were not sensitive to the allocation of medical resources, and other factors still need to be solved.

**Table 6:** Time series regression results of accumulated medical materials.

Variables	MPC	N95	MSM	MPFS	MIG
Wuhan	−8.49^$^	3.29	1.44^&^	−0.02	0.53^&^
Xiangyang	−0.31	0.35	−0.39^$^	0.30	−0.16
Yichang	−1.38^#^	−0.40	0.40^#^	2.93^$^	−1.69^$^
Huangshi	0.69^#^	0.66	0.23^#^	1	0.57^&^
Shiyan	2.05^&^	−1.83^&^	0.5^#^	−0.39	−0.72^$^
Jingzhou	0.62	−1.39^#^	−0.08	−0.11	0.44
Jingmen	1.08^#^	−1.35	0.38	−0.28	−0.24
Ezhou	−1.48^#^	2.02^#^	0.31	−0.71	−0.65
Xiaogan	−0.79	−1.99	0.42	0.45	0.77
Huanggang	1.04	0.48	−0.3#	−1.4	−0.06
Xianning	0.36^#^	0.01	−0.28^&^	−1.4^&^	1.19^&^
Suizhou	0.40	−1.70^&^	−0.07	0.58^#^	0.39
Enshi	0.52	−0.03	−0.11	−0.79	0.22
Xiantao	−1.45^$^	1.47	−0.38	−0.46	0.61
Tianmen	−0.45	0.59	−0.38^$^	0.87^$^	−0.72
Qianjiang	0.29	−1.67^&^	0.18^#^	1.33^&^	−0.37^#^
Shennongjia	0.05	−0.24	−0.18^&^	0.06	0.37^$^

Significant codes: 0 &, 0.001 ^, 0.01 $, 0.05 #, 0.10.

The new cases in Wuhan, Yichang, Shiyan, Ezhou, and Xiantao decreased significantly with the increase of MPC; The number of new cases in Shiyan, Jingzhou, Suizhou, and Qianjiang decreased significantly due to N95; MSM had a significant effect on new cases such as Xiangyang, Huanggang, Xianning, Tianmen, and Shennongjia; Xianning was significantly affected by MPFS; The influential factor in new cases in Yichang, Shiyan, and Qianjiang was MIG.

It was not difficult to find that the accretion of medical factors would mostly lead to the reduction of new cases. However, we may also see that the increase in medical factors will mainly lead to the reduction of new cases, which indicated that there are still problems in the supply of medical materials. On March 6, 2020, Sohu News released that the gap in medical materials in Hubei is still large (20). Our research results also reflect this situation. For example, in Huangshi, Jingmen, Xianning, and other places, MPC materials are seriously insufficient, and new cases still increase significantly.

### 3.3. Comparison with Guangdong Province

In order to explore the impact of social factors on COVID-19 in other regions, this paper selects Guangdong Province for a comparative study. The research method is the same as above. The independent variable is removed from the distance, and x1, x2, x3, and x5 are selected to represent the number of university, population, the number of hospital, GDP. Regression results were as shown in Table 7.

**Table 7:** Regression result data of Guangdong Province.

Variables	T	T/P	A	A/P
University	0.043	0.519	−0.235^^^	−0.112
Population	−4.66^^^	−1.64	−0.523^^^	−1.026
Hospital/p	0.079	−0.79	0.186^#^	−0.098
GDP	1.32^&^	1.56^^^	1.457^&^	1.742^&^

Significant codes: 0 &, 0.001 ^, 0.01 $, 0.05 #, 0.10.

The comparison showed that the GDP of Guangdong Province has a significant impact on the total number of cases and the proportion of new cases. The reason is that the higher the GDP, the better the economic situation of the region, the more economic exchanges, the higher the risk of infection, which will lead to an increase in the number of cases. In addition, the more universities and population in Guangdong Province, the fewer new cases, indicating that the prevention and control measures in Guangdong Province effectively controlled the spread of COVID-19 at the beginning of the epidemic. By the way, new research showed that in (15), while Guangdong successfully controlled the COVID-19 with Non Drug Interventions (NPI), it also “accidentally” achieved the containment effect of 39 other statutory infectious diseases, with the total number of cases falling 65.6% from the expected level, reducing the incidence of nearly one million people (17).

The comparison between Guangdong Province and Hubei Province showed that social factors will have an impact on COVID-19, but due to regional differences, the degree of impact is also different.

### 3.4. Lag period

For further exploration, this paper used the Almon polynomial to study the lag effect of time series of medical materials on the number of new cases. The areas with lag period were shown in Table 8.

**Table 8:** Region and its lag period.

Region	Lag period (day)
Wuhan	1
Jingmen	3
Suizhou	4
Qianjiang	3

## 4. Discussion

We may expect that the high density of population and universities means more personnel mobility, which was the main way of virus transmission. In addition, the farther the distance, the fewer confirmed cases. The more medical supplies, the fewer new cases. However, the results showed that the impact of the number of hospitals was not important. In addition, the distribution of some medical materials had a very different impact on the number of new cases in each city. With the accretion of materials, the number of new cases gradually decreased. However, the data showed that the supply of medical materials in some cities is insufficient, and the number of new cases was still increasing significantly. Another explanation may be that with the increase in the supply of medical surgical masks, the burden of medical waste increased, and improper treatment of medical materials led to more infections. To sum up, we found that the evolution of COVID-19 was affected by some social factors, which may help in healthcare policy-making. At the same time, the rational distribution of medical materials must be ensured.

## 5. Conclusion

This study investigated the social factors affecting confirmed cases of COVID-19 in 17 cities of Hubei Province. We found that the city could be divided into three groups according to the number of confirmed cases and the time course of cases. According to the research results, some suggestions of public health policy could be put forward.

For medical materials, this paper put forward the following suggestions: in order to carry out the prevention and control of COVID-19, the rational allocation of various medical materials would be of great benefit, but we also need to pay attention to improving protective measures in hospital, doing a good job in isolation and preventing accidents. Irregular handling of medical materials could also lead to infection. Hospital managers should properly deal with medical waste by improving the quality of employees. At the same time, the supply of medical materials should also consider their lag effects, reduce the waste of medical materials and ensure the maximization of the utility of materials. Therefore, it was particularly important to understand the supply, transportation, and treatment of medical materials.

From the perspective of the number of “population” and “universities,” the suggestions put forward in this paper were as follows: first, do a good job in the management and control of colleges and universities, and reduce the flow of students outside winter vacation and summer vacation. Second, promote the construction of medical schools. At present, there were few independent medical schools. The development of most urban medical schools depended on the existing local comprehensive universities. The establishment of medical colleges and departments and medical schools could greatly improve the local medical resources and strength. For some cities with a large population, especially Huanggang City, which had a registered population of more than 5 million, there were only 21 hospitals. Its medical strength was quite weak and needs to be strengthened urgently. For some small and medium-sized cities in Wuhan city circle, it was very important to rely on Wuhan’s rich medical college resources, strengthen cooperation, speed up construction and improve medical capacity.

## Data availability statement

The original contributions presented in the study are included in the article/Supplementary material, further inquiries can be directed to the corresponding author.

## Author contributions

SY and LD: conceptualization and validation. SY: methodology and writing—original draft preparation. LD: resources, writing—review and editing, supervision, project administration, and funding acquisition. DM: data collection and collation. All authors have read and agreed to the published version of the manuscript.

## Funding

This research was funded by the National Natural Science Foundation of China, grant numbers 72104190 and 72042015; Humanities and Social Sciences Youth Foundation, Ministry of Education of the People’s Republic of China, grant number 20YJC630018.

## Conflict of interest

The authors declare that the research was conducted in the absence of any commercial or financial relationships that could be construed as a potential conflict of interest.

## Publisher’s note

All claims expressed in this article are solely those of the authors and do not necessarily represent those of their affiliated organizations, or those of the publisher, the editors and the reviewers. Any product that may be evaluated in this article, or claim that may be made by its manufacturer, is not guaranteed or endorsed by the publisher.

## Figures and Tables

**Figure A1:**
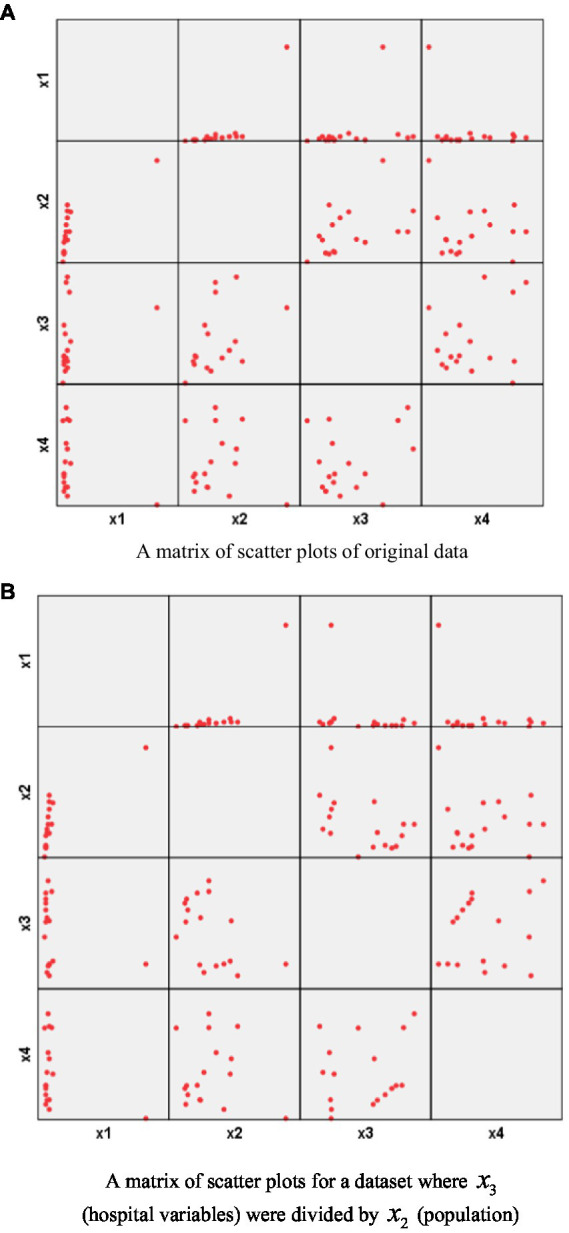
**(A)** A matrix of scatter plots of original data. **(B)** A matrix of scatter plots for a dataset where $x_{3}$ (hospital variables) were divided by $x_{2}$ (population).

## Footnotes

^1^ https://news.qq.com/zt2020/page/feiyan.htm#/

^2^ https://www.hubei.gov.cn/

^3^ http://tjj.hubei.gov.cn/
